# Supplementary material for: A Review and a Framework of Variables for Defining and Characterizing Tinnitus Subphenotypes
Source: Brain Sci. 2020 Dec 4;10(12):938. doi: 10.3390/brainsci10120938 (PMC7762072; doi:10.3390/brainsci10120938)
Supplement: Supplementary file 1 [file brainsci-10-00938-s001.zip › Supplementary Material Table S1.rtf]

Table S1. Non tinnitus-specific variables.
Subdomain	Variable Concept	Assessed (n studies)	Used for subgrouping (n studies)	Significantly differing or important for classification (n studies)	
Demographic and Socio-economic Characteristics	Age	43	2	21	
	Sex	38	3	13	
	Employment and Occupation	10	0	5	
	Education	10	0	4	
	Marital Status	10	0	3	
	Hearing-related Family History	5	0	4	
	Economic Status	4	0	3	
	Ethnicity and Nationality	3	0	1	
	Handedness	3	0	0	
Lifestyle and Exposures	Physical Activity	2	0	2	
	Substance Use	2	0	1	
	Noise Exposure	1	0	0	
	Smoking	1	0	0	
Ear and Hearing Function	Hearing Ability	43	8	15	
	Problems With Sounds	19	6	7	
	Hearing Aid Use	7	0	1	
	Various Ear Conditions	3	0	1	
	Vertigo	2	0	0	
	Preference for Environmental Sound	1	1	0	
	Reaction to Acoustic Stimuli	1	0	1	
Mental Health	Depressive Symptoms	32	4	19	
	Symptoms of Anxiety	20	2	12	
	Stress-related Symptoms	12	1	8	
	Personality and Coping Strategies	11	3	5	
	Various Psychological Conditions*	8	3	5	
	Mood	2	1	1	
Other Symptoms and Conditions	Various Pain Problems	15	2	10	
	Headache	14	3	6	
	Dizziness	14	1	10	
	Mandible Problems	13	4	6	
	Quality of Life	12	0	11	
	Neck Problems	11	0	9	
	Various Symptoms and Conditions	10	1	5	
	Various Somatic Symptoms	8	4	4	
	Sleep Problems	6	0	1	
	Various Head and Neck Problems	4	0	1	
	Circulatory System Problems	3	1	0	
	Cognitive Functions	3	0	1	
	Endocrine, Nutritional and Metabolic Problems	3	0	1	
	Symptoms of Fatigue	2	0	1	
	Vision Problems	1	1	0	
	Mobility Problems	1	0	1	
	Nervous System Problems	1	0	1	
	Physical Trauma	1	0	0	
Healthcare and Treatments	Psychiatric Treatments	10	0	7	
	ENT Stays	1	0	1	
Brain Anatomy and Function	Activation Patterns	7	1	5	
	Connectivity	3	0	3	
	Structural Features	2	1	1	
Genetic Profile	Single Nucleotide Polymorphisms	2	1	1	
Note: If a variable was used more than once in a study it's most significant contribution was considered in counts (used for subgrouping > significantly differing or important for classification > not important for subphenotyping). 
*Includes combined variable of depression and anxiety, and other conditions such as obsessive compulsive symptomatology.
