# Supplementary material for: A Review and a Framework of Variables for Defining and Characterizing Tinnitus Subphenotypes
Source: Brain Sci. 2020 Dec 4;10(12):938. doi: 10.3390/brainsci10120938 (PMC7762072; doi:10.3390/brainsci10120938)
Supplement: Supplementary file 1 [file brainsci-10-00938-s001.zip › Supplementary Material Table S2.rtf]

Table S2. Tinnitus-specific variables.
Subdomain	Variable Concept	Assessed (n studies)	Used for subgrouping (n studies)	Significantly differing or important for classification (n studies)	
Onset Related Characteristics	Duration	37	3	9	
	Gradual or Abrupt Onset	12	1	3	
	Temporal Relationship with Other Incidence	10	1	5	
	Age at Onset	8	2	5	
	Emergence Time of Day	3	0	2	
	Trigger Beliefs*	1	0	1	
Perceptual Characteristics	Loudness	28	3	11	
	Localisation	27	4	6	
	Pitch	23	3	5	
	Quality	19	3	7	
	Varying Perception	13	2	7	
	Rhythmicity	13	0	6	
	Presence Pattern†	12	3	3	
	Number of Sounds	1	0	0	
Modulating Factors	External Sound Effect	21	3	12	
	Somatic Manoeuvres Effect	16	4	7	
	Psychological Factors Effect	10	2	5	
	Sleep Effect	7	1	4	
	Medication Effect	4	0	2	
	Other Sensory Stimulation Effect	1	1	0	
	Physical Activity Effect	1	0	1	
	Easing Factors‡	1	0	0	
	Substances Effect	1	0	0	
Associations with Other Conditions	Tinnitus Pitch and Hearing Loss Profile	3	0	1	
	Tinnitus Localisation and Hearing Loss Profile	2	1	1	
	Tinnitus Localisation and Vascular Comorbidities	1	0	1	
	Somatic Manoeuvres Effect on Tinnitus and Somatic Disorders	1	0	1	
Impact and Reactions	Overall Severity	54	18	20	
	Impact on Emotion and Mental Health	25	2	13	
	Awareness	9	0	3	
	Intrusiveness and Ability to Ignore	8	0	2	
	Impact on Sleep	7	1	2	
	Impact on Concentration	6	1	2	
	Impact on Hearing	3	1	2	
	Ability to Cope	2	1	1	
	Impact on Situations	2	0	2	
	Impact on Somatic Sensations	2	0	2	
	Acceptance	2	0	1	
Healthcare and Treatments for Tinnitus	Clinician Consultation	8	1	4	
	Preceding Tinnitus Treatments	7	0	5	
	Treatment Response	5	2	3	
Clinical Subtypes	Somatosensory Tinnitus	3	3	0	
	Typewriter or Middle Ear Myoclonus Tinnitus	1	1	0	
	Likely Tinnitus Aetiology	1	1	0	

Note: If a variable was used more than once in a study it's most significant contribution was considered in counts (used for subgrouping > significantly differing or important for classification > not important for subphenotyping). 
*Patient report of what they believe is the main trigger for tinnitus; †Patient's description of the presence of tinnitus during a day e.g. whether it is constantly present or intermittent; ‡Patient's report of the possibility to do anything to lessen tinnitus-related problems, or situations when it is less problematic.
